# Circular Bioassay Platforms for Applications in Microwave-Accelerated Techniques

**DOI:** 10.5101/nbe.v6i4.p85-93

**Published:** 2014-12-02

**Authors:** Muzaffer Mohammed, Travis C. Clement, Kadir Aslan

**Affiliations:** Morgan State University, Department of Chemistry, Baltimore MD 21251

**Keywords:** Circular PMMA platform, COMSOL simulations, Microwave-accelerated bioassays, Silver island films, Microwave heating, Metal-assisted and microwave-accelerated evaporative crystallization

## Abstract

In this paper, we present the design of four different circular bioassay platforms, which are suitable for homogeneous microwave heating, using theoretical calculations (i.e., COMSOL™ multiphysics software). Circular bioassay platforms are constructed from poly(methyl methacrylate) (PMMA) for optical transparency between 400–800 nm, has multiple sample capacity (12, 16, 19 and 21 wells) and modified with silver nanoparticle films (SNFs) to be used in microwave-accelerated bioassays (MABs). In addition, a small monomode microwave cavity, which can be operated with an external microwave generator (100 W), for use with the bioassay platforms in MABs is also developed. Our design parameters for the circular bioassay platforms and monomode microwave cavity during microwave heating were: (i) temperature profiles, (ii) electric field distributions, (iii) location of the circular bioassay platforms inside the microwave cavity, and (iv) design and number of wells on the circular bioassay platforms. We have also carried out additional simulations to assess the use of circular bioassay platforms in a conventional kitchen microwave oven (e.g., 900 W). Our results show that the location of the circular bioassay platforms in the microwave cavity was predicted to have a significant effect on the homogeneous heating of these platforms. The 21-well circular bioassay platform design in our monomode microwave cavity was predicted to offer a homogeneous heating pattern, where inter-well temperature was observed to be in between 23.72–24.13°C and intra-well temperature difference was less than 0.21°C for 60 seconds of microwave heating, which was also verified experimentally.

## Introduction

Microwave heating has found its way into a wide range of applications in various disciplines, including food industry [1–3], chemical synthesis [4, 5], drug discovery [6, 7], sterilization (bactericidal and fungicidal) [8, 9] and rapid bioassays [10–14]. Most common form of microwave heating is the dielectric heating, which can be described as the distribution of heat generated by molecular friction created as a result of molecules attempting to align themselves with the oscillating electric field [1, 15]. In this regard, dielectric heating has been routinely applied to chemical synthesis, where an increase in the rate of product formation is observed due to microwave heating [5].

In the field of rapid bioassays, the concurrent use of microwave heating and plasmonic nanoparticles was demonstrated to significantly reduce assay times, which was attributed to microwave-induced temperature gradients generated between the bulk and the plasmonic nanoparticles [12, 16–20]. The combined use of microwave heating and plasmonic nanoparticles was also demonstrated for rapid evaporative crystallization of amino acids and other small molecules, (i.e., metal-assisted and microwave-accelerated evaporative crystallization, MA-MAEC) [21–26].

Despite the advantage of rapid processing times afforded by microwave heating, heterogeneous heating of the system of interest comprised of components with different dielectric properties is a major concern for microwave heating based applications [15, 27, 28]. Various solutions were proposed to alleviate the issue of heterogeneous heating, including the use of turntables and mode stirrers [29], modifications to the geometry of target, the use of low power microwaves for longer duration, variations of the thickness of the target to ensure deeper penetration of microwaves [15]. However, these solutions are ineffective in solving the heterogeneous heating issue for the emerging new technologies, such as MA-MAEC and MABs.

In both MA-MAEC and MABs, currently available tools (microscope slides and high throughput screening (HTS) microplates) are ill-suited for microwave heating: they contain right-angle corners that reflect/absorb the electric field resulting in heterogeneous heating. Subsequently, there is still a need to develop new tools for MA-MAEC and MABs by specifically designing them for homogeneous microwave heating of samples, where the use of microwave heating can have significant advantages over traditional methods [12, 21].

In this work, we present the results of our theoretical investigation of the design of two new tools for MA-MAEC and MAB: 1) new circular bioassay platforms with multiple sample capacity and 2) a monomode microwave cavity that is specifically designed for the homogeneous heating of new circular bioassay platforms. Circular bioassay platforms are designed to have the following advantages over the currently available tools: i) have smooth edges to prevent the electric field to be focused, ii) dimensions (i.e, 5 cm in diameter) that is smaller than the wavelength of microwaves at 2.45 GHz (ca. 12.2 cm) to minimize their interactions with the microwaves, and iii) covered with a silicon isolator with various number of wells for multiplexed processes, which help in the reduction of excess heat. In addition, using theoretical simulations, a small monomode microwave cavity that works with a 100 W external solid state microwave generator was developed for homogeneous heating of the circular bioassay platforms.

Our theoretical simulations include electric field and temperature distributions for the circular bioassay platforms placed in our microwave cavity and a conventional kitchen mincrowave oven. In addition, we have constructed a 21-well circular bioassay platform and the monomode microwave cavity and provided experimental real time temperature profiles of a circular bioassay platform heated in our monomode microwave cavity. We envision that these new biosensing systems will be useful for environmental sensing, rapid diagnostic applications based on MABs technique and MA-MAEC technique.

## Methods and materials

### Theoretical simulations

COMSOL™ multiphysics software (version 4.3b) was used to simulate the microwave heating of HTS microplates and circular bioassay platforms in both conventional microwave oven and in our monomode microwave cavity. COMSOL™ software includes an inbuilt microwave heating model for a conventional microwave oven, which was modified to simulate electric field and temperature distributions around our circular bioassay platforms when placed in a conventional microwave oven and inside our monomode microwave cavity. Boundary conditions and study designs were kept identical to the inbuilt simulation model. Table 1 lists the parameters used in simulations for both conventional microwave oven and small microwave cavity. Water was used as a target sample for microwave heating as all assay steps involve use of water based buffers. Calculations pertaining to microwave heating are described by Maxwell’s equations and have been previously published [2, 3, 15]. A frequency transient study with time dependent solver (5 sec steps) was conducted for complete microwave cavity rather than for a symmetric half. Finite difference time domain (FDTD) method was used in computation of equations. Heat transfer study module of COMSOL™ multiphysics software is also use to predict the heat distribution across wells during microwave heating steps.

### Microwave systems and platforms

**Fig. 1:**
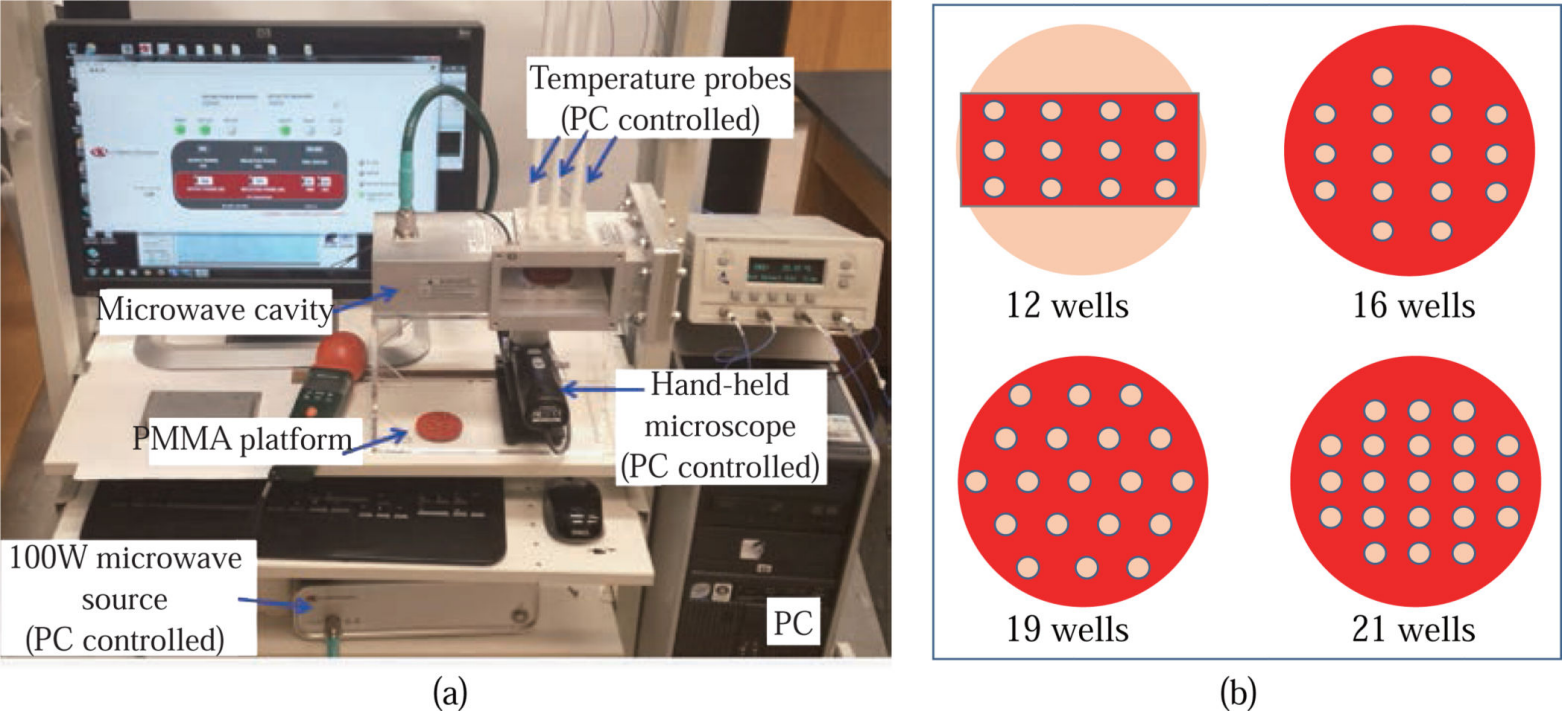
(a) Our microwave cavity with an 100 W external microwave generator and temperature probes. (b) Different circular bioassay platform designs used for simulations.

Figure 1 shows images of the small microwave cavity and the platforms used in the study. For simulations of a conventional microwave oven, dimensions of 900 W conventional microwave oven (Frigidaire, Model No: FCM09Z03KB) were used including the location of waveguide and the turn table. The small microwave cavity was based on WR-430 waveguide dimensions having a frequency match range between 1.7–2.6 GHz and powered by Emblation ISYS connect microwave generator that produces 100 W variable power at 2.45 GHz, transmitted to the cavity using coaxial to waveguide adapter. Length of the chamber was 20 cm excluding the length of adapter which is 11 cm in length. The materials for our microwave cavity was aluminum (type: 6061-T6) and the simulations included circular bioassay platforms (diameter = 5 cm) with silicon isolators. Four well designs of the bioassay platforms with 12, 16, 19 and 21 wells were investigated for homogeneous heating. All material properties were obtained from COMSOL™ material library and were kept consistent for both microwave systems. One of the openings of the microwave cavity was selected as a wave port with previously described power and impedance settings. Temperature trend of each well was recorded through color coding scale of COMSOL™ at every 5 sec step of simulation.

### Experimental temperature measurements

Real time temperature measurements were carried out using a FISO multichannel fiber optic sensor (Fig. 1) in our monomode microwave cavity for 5 min (followed by 10 min of cooling). Two 21-well circular bioassay platforms coated with 10 nm silver nanoparticle films were prepared according to method described previously [12]. All wells were filled with 30 μl deionized water. In our monomode microwave cavity, four fiber optic sensor cables were used; one kept at room temperature outside the chamber, second in extreme left well from center, third in extreme right well of the center and fourth was suspended in the chamber to monitor the temperature of the chamber. Data was plotted using Sigma Plot 11.2 statistics software.

## Results

**Fig. 2:**
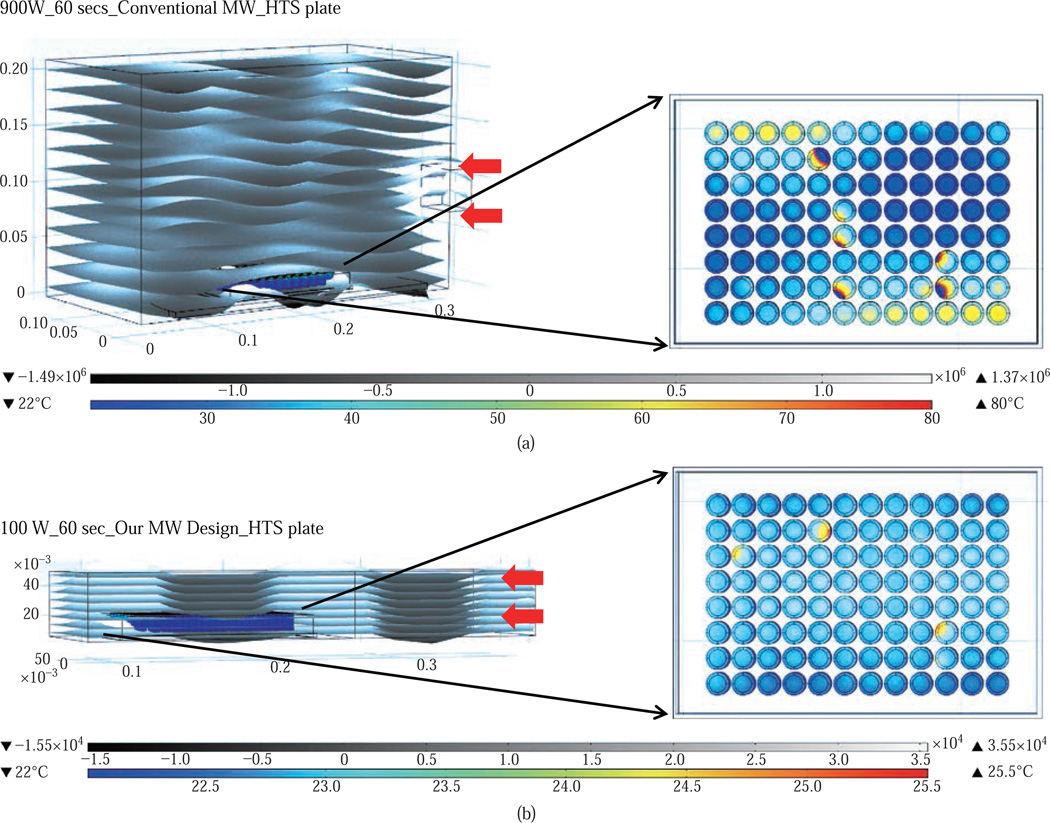
Heating pattern of a 96 wells HTS microplate in a 900 W conventional kitchen microwave oven and our monomode microwave cavity.

Figure 2 shows the results of the simulated electric field distribution and the predicted temperature of the HTS microplates in a 900 W conventional microwave oven and in our monomode microwave cavity. It is important to mention that the surface and sides of HTS microplates are not shown to visually differentiate the temperature of the wells. In both microwave cavities, HTS microplates were predicted to be heated in a heterogeneous fashion, which can be deduced from the comparison of the predicted temperature values of each well. That is, simulated electric field inside the conventional microwave oven shows two hot spots (Fig. 2(a)-inset, (i) Left-top and (ii) Bottom-right of the HTS microplates). This prediction can be attributed to the size of the HTS microplates, which is comparable to the wavelength of the microwaves at 2.45 GHz (ca. 12.2 cm). The temperature range of HTS microplates is predicted to be between 30–80°C after 60 sec of heating. As expected, the lower temperatures are predicted to be outside the hot spots and the higher temperatures are predicted to be inside the hotspots. The heterogeneous heating can also be contributed by the multimode nature of the conventional microwave oven, where several electromagnetic transmission modes propagate simultaneously resulting in interference effect and inconsistent hot spots inside the cavity.

In our small monomode cavity, where the simulations are carried out for 60 sec of microwave heating at 100 W, HTS microplates are also predicted to be heated in a heterogeneous manner, as shown in Fig. 2(b). The temperature range of the HTS microplates varies between 22.6 and 25.5°C. The temperature of the wells in the mid-section of the HTS microplate is predicted to be 22.9–23.1°C, which larger than the temperature of the wells (<22.5°C) located closer to the edges. These results imply that HTS microplates are incompatible with MABs technique and the resulting heterogeneous heating will adversely impact the various steps of experiments in which microwave heating is used thereby altering the outcome of the experiment.

**Fig. 3:**
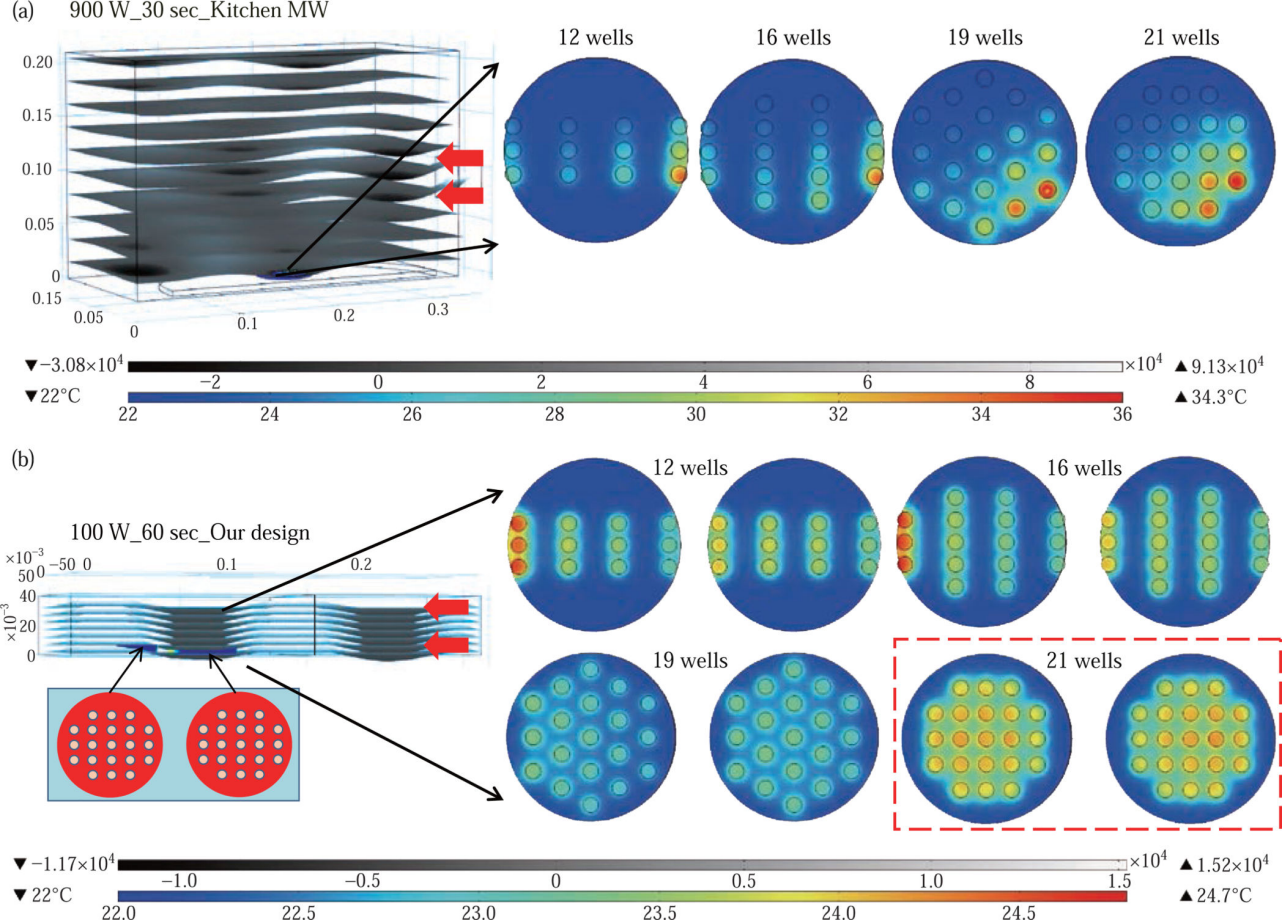
(a) Electric field pattern in a 900 W kitchen microwave and heating pattern of different well designs on circular bioassay platform. (b) Heating pattern of different well designs on circular bioassay platform in our 100 W monomode microwave cavity. The red arrows show the source and the direction of the microwaves.

Based on these initial results, we carried out simulations for our circular bioassay platforms in a conventional microwave oven and in our monomode small microwave cavity. Figure 3(a) shows the temperature profiles and electric field distributions for our circular bioassays platforms in a 900 W conventional microwave oven for 30 sec. Our criteria for the selection of microwave heating time was based on the requirement for the assay temperature < 37°C to prevent the denaturation of proteins. We have also investigated microwave heating up to 60 seconds, which proved that the temperature of the wells is predicted to be significantly larger than 37°C (data not shown for brevity of the work). It is also important to note that the presence of a turntable and its rotation cannot be simulated using COMSOL multiphysics software; thereby the results reported are for a static circular bioassay platform with no rotation and movement in the cavity. The location of our circular bioassay platforms was varied in our simulations in order to determine the best location inside the microwave cavity for possible homogeneous heating. We have also carried out simulations for one, two, three and four circular bioassay platforms placed multiple combinations of locations inside the conventional microwave cavity and these simulations showed heterogeneous heating across the wells (data not shown).

After 30 sec of heating in the conventional microwave oven, all well designs show heterogeneous heating across all wells with temperature ranging from 27–36 °C for 12-well, 26.5–34.7°C for 16-well, 22–34.1°C for 19-well and 23–35.2°C for 21-well design. The significant temperature differences between wells on same platforms can be attributed to multiple modes of EM propagation in the conventional kitchen microwave ovens, which result in heterogeneous heating of objects across different regions. Figure S1 shows the predicted temperature vs time vs well number plots of 12-, 16-, 19- and 21-well platform in a 900 W conventional microwave oven. It is observed that each well shows a different temperature at any given point and the temperature variation between each well increases with heating time. However, with the inclusion of turntable in the actual experiments, the heterogeneous heating can be reduced due to the rotation of the turntable and the interference patterns of multiple EM modes in the microwave cavity [1, 15].

Figure 3(b) shows the electric field distribution in the small monomode microwave cavity with a 100 W external microwave generator. Simulations were conducted for one and two circular bioassay platforms in the small microwave cavity. We note that our small microwave cavity can only hold a maximum of two circular bioassay platforms at the same time. The location of the platforms was optimized with respect to the E-field in order to achieve maximum homogeneous heating across all wells. In Figure 3B-Inset shows the predicted temperature profiles of the two circular bioassay platforms for all four well designs. The 12- and 16-well designs are predicted to have significant variation in the temperature of the wells, where higher temperatures are predicted for the wells further away from the microwave source. On the other hand, the simulations for 19- and 21-well platforms show a more homogeneous temperature distribution across all wells.

Figure S2 shows the predicted temperature profiles of all wells of the two circular bioassay platforms for all four well designs during microwave heating in our monomode microwave cavity. The significant variation in temperature of wells is clearly seen for 12-, 16- and 19-well platforms, where the variation in temperature of the wells in 21-well design was minimal. After 60 sec of heating in our small microwave cavity, the temperature range for 12- and 16-well platforms is predicted to be 22.5–24.7°C and 23.1–24.9°C, respectively. The overall temperature range for wells in both 19-well platforms is 22.6–23.6°C. The 21-well design shows the most homogeneous temperature distribution with almost all the wells are predicted to have the same temperature. The temperature range for wells on the two 21-well platforms was observed to be in between 23.72–24.13°C. Although not investigated here, it should be noted that the significant variation in temperature of the wells can negatively impact the outcome of MABs.

**Fig. 4:**
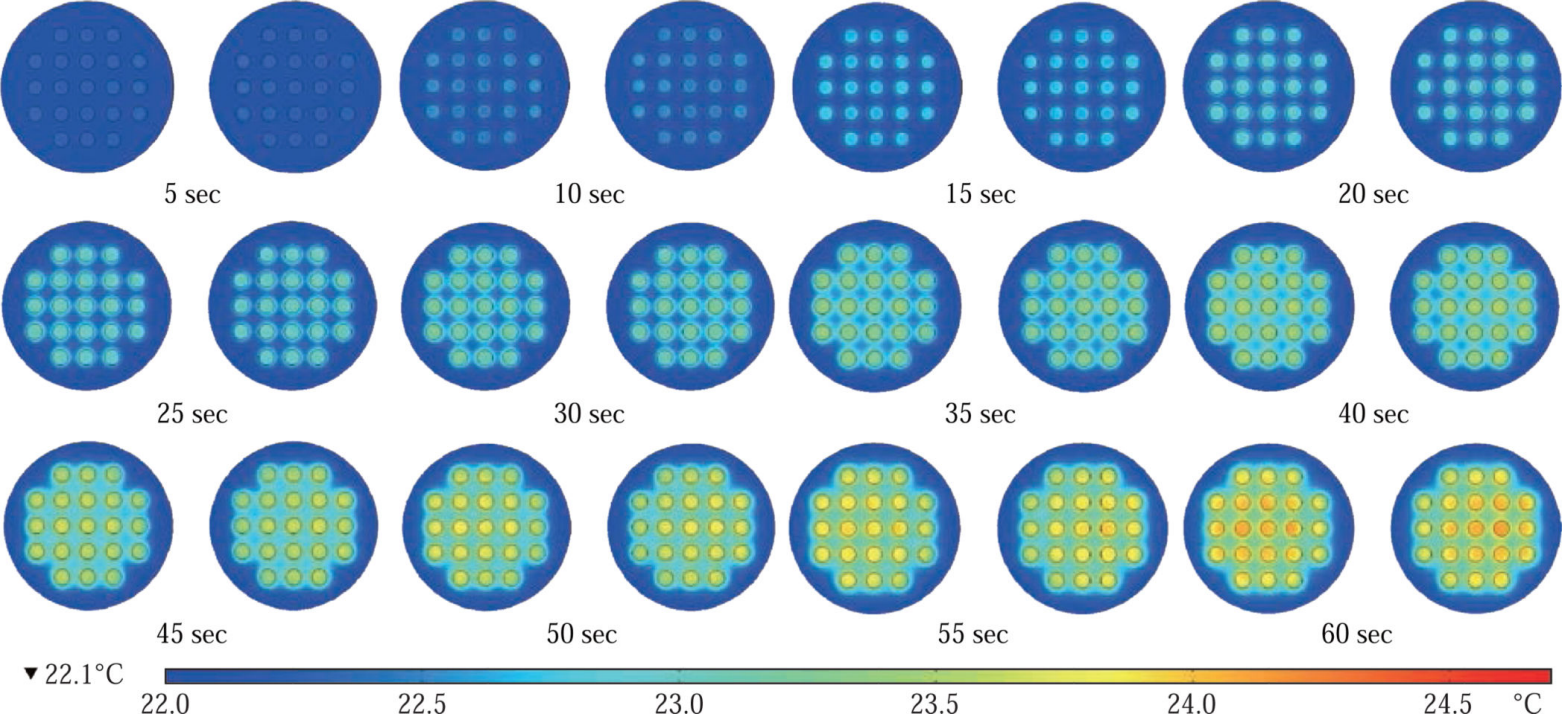
Timed images of heating profile of two 21-well circular bioassay platform in our monomode microwave cavity using 100 W power for 60 sec.

Figure 4 shows the simulated images of 21-well platforms during microwave heating in small microwave cavity at 5 sec interval up to 60 sec. These simulations yielded that the temperature increase in the 21-well platforms during microwave heating follows a linear trend for all the wells and the temperature variation between wells at any time of microwave heating remain relatively small. However, some of the wells of 21-well platform closer to the microwave source show a higher temperature increase as compared to the other wells, which can be attributed to their location between the negative and positive amplitude of the microwaves. However, the variation in temperature was < 0.18°C, which is not expected to have significant impact on the outcome of the MABs.

**Fig. 5:**
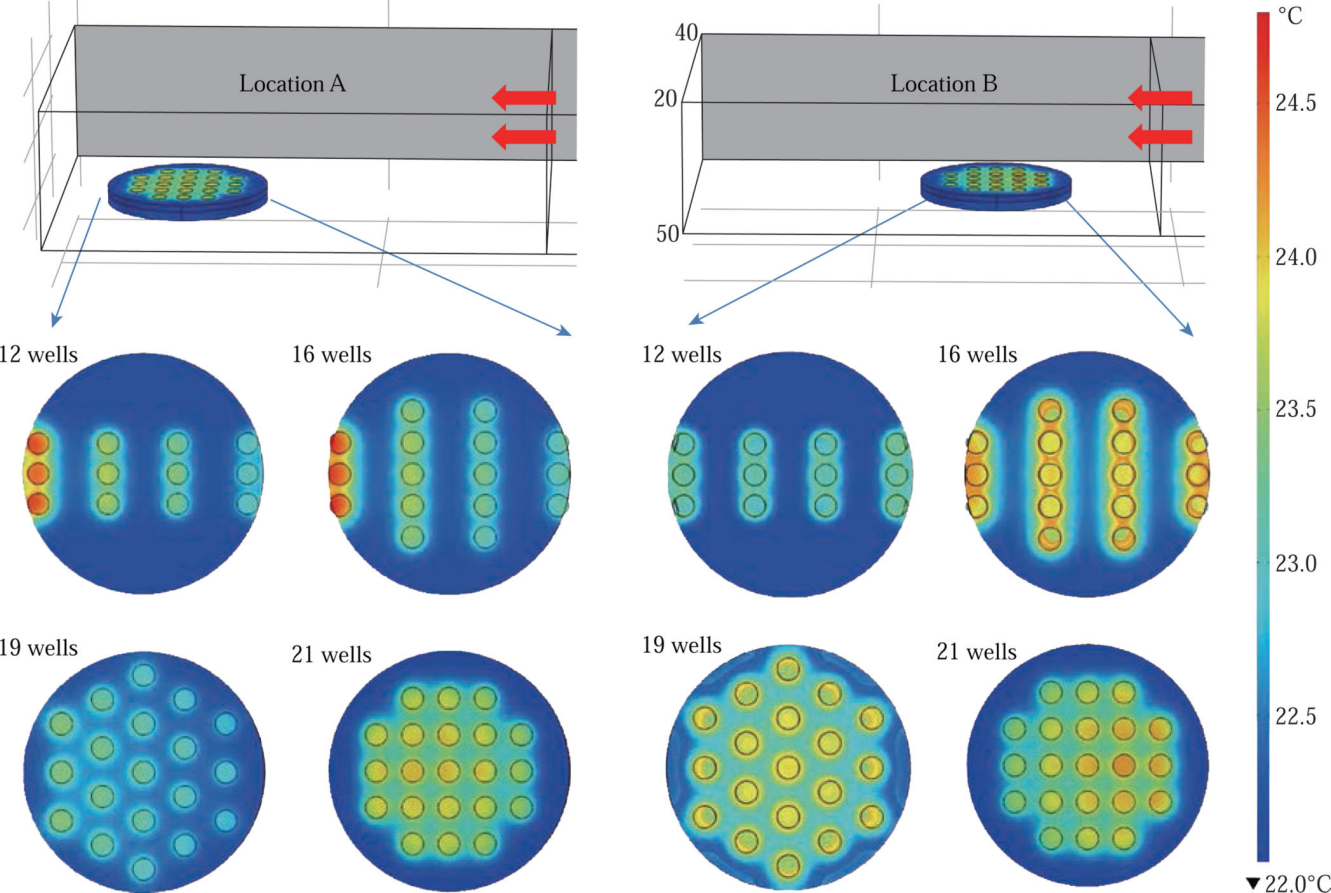
Heating pattern of a one circular bioassay platform at different locations in monomode microwave cavity after 60 sec of microwave heating. The red arrows show the source and the direction of the microwaves.

Figure 5 shows the individual simulated temperature profiles for four different circular bioassay platforms after microwave heating in our small monomode cavity for 60 sec. These studies were carried out for only one circular bioassay platform at a time in monomode cavity and these platforms were placed at two different locations within the cavity: Location A and Location B. At location A, all four circular bioassay platforms are predicted to have significant variation in temperature of the wells, after 60 sec of microwave heating. The wells of the 12-, 16- and 19-well platforms located further away from the microwave source show ~2°C (24.6°C) higher temperature as compared to the wells located closer to the source (22.7°C). On the other hand, 21-well platform shows homogeneous temperature distribution across all 21 wells, with a slight variation in temperature (~0.2°C) within some wells closer to the microwave source. At location B, all four circular bioassay platforms are predicted to be heated in a more homogeneous manner as compared to the platforms placed at location A. Wells located closer to the microwave source are predicted to have higher temperature as compared to wells located further away from microwave source. Temperature range for 12-, 16- and 19-well platforms are found to be 23.1 and 24.3°C with average intra-well temperature difference of 0.3–0.5°C. The temperature of the 21-well platform is predicted to be 23.7–24.1°C with an intra-well temperature difference of 0.16°C. Higher temperatures of wells at location A as compared to those at location B is attributed to the presence of hot spot encompassing the entire platform and also in part to subsequent reflection of microwave energy from shortening plate present closer to location A. Figure S3 and S4 show the predicted temperature increase for 21-well platforms during microwave heating for 60 sec at locations A and B. At both locations, a linear increase in temperature was observed across all 21 wells during 60 sec of microwave heating. The inter-well temperature difference at location A was lower as compared to wells of the circular bioassay platforms at location B, indicating location A as ideal for microwave heating of single 21-well platform.

In order to verify that the temperature values predicted by theoretical simulations (in Figure S2D) can be obtained experimentally, real time temperature of selected wells on 21-well platforms was carried out during microwave heating in our small microwave cavity using optic fiber sensors and shown in Figure S5. The locations of the observation ports on small microwave cavity facilitated temperature measurements for selected wells on a single circular bioassay platform. We note that the predicted temperature for all wells of two 21-well circular bioassay platform (Figure S2D) implied homogeneous heating of all wells and a simultaneous temperature increase with each heating steps. The difference in lowest and highest temperature predicted by simulations in all 42 wells was <0.4°C. Real time temperature values in the selected wells of a 21 well platform (shown in Figure S5) were found to be similar to the temperature values predicted by simulations. Temperature of microwave cavity was observed to increase with the initiation of microwave heating and reached 33.7°C after 2 min, remaining almost constant throughout the heating period. After the microwave energy was turned off, the temperature of the microwave cavity decreased to 21.8°C at the end of cooling period. Wells selected for this experiments (highlighted in Figure S5 by arrows) show proportional increase in temperature from initial value of 22.3°C to a maximum value of 24.15°C at the end of heating period. It is important to note that the difference in temperature between the two wells, which was found to be 0.22°C (within the acceptable error in the calibration of these sensors) remains almost constant throughout the period when microwave energy was supplied. Eventually, both wells reach to the same temperature at the end of cooling period 10 min after the microwave energy is turned off. These observations imply that the 21-well circular bioassay platform design is ideal for homogeneous heating, which is critical to the success of applications of microwave-accelerated bioassays.

Our future work includes the development of a new 10 cm circular bioassay platform with 95 and 204 sample well capacities, which will be an alternate platform to the traditional 96 well HTS microplates, for microwave heating applications. The sample size for 95 wells will be 30 μl and for 204 wells will be 10 μl. Both these platforms will work exclusively with the small monomode microwave cavity, which various detection (colorimetric, fluorescence and chemiluminescence) probes, fiber optic temperature probe and optical imaging camera as a single self-contained system with multiple sample capacity for laboratory scale experiments and biosensing applications. These reports will be released in due course.

## Conclusions

Using theoretical simulations and experimental work, we demonstrated that our new circular bioassay platforms afford for homogeneous heating across all wells during the microwave heating. We have also shown the advantage of using a monomode small microwave cavity for heating small samples. With the small monomode microwave cavity and new 21-well circular bioassay platforms, a better control over the microwave heating steps of the bioassays can be achieved for the prevention of the generation of false data, and the speed and efficiency of bioassays can increased. The small microwave cavity is light weight and portable, making it ideal for on-site environmental sensing applications [12], rapid small scale crystallization of amino acids and proteins.

## Supplementary Material

supporting

## Figures and Tables

**Table 1:** Parameters used for simulation of 900 W conventional kitchen microwave and our monomode microwave cavity

Parameter	900 W commercial	100 W small chamber
Power	900 Watts	100 Watts
Frequency	2.45 GHz	2.45 GHz
Impedance	50 Ω	50 Ω
Sample	Water	Water
Initial temperaturec	22 °C	22 °C
Time of heating	30 sec	60 sec

## Abbreviations

MABs: Microwave-accelerated bioassays
PMMA: poly(methyl methacrylate)
MA-MAEC: Metal-assisted and microwave-accelerated evaporative crystallization
HTS: High throughput screening microplates
