# Supplementary material for: Circular Bioassay Platforms for Applications in Microwave-Accelerated Techniques
Source: Nano Biomed Eng. Author manuscript; Available in PMC 2015 Jan 5. (PMC4283778; doi:10.5101/nbe.v6i4.p85-93)
Supplement: supporting [file NIHMS648360-supplement-supporting.docx]

**Supporting Information for**

**“Circular Bioassay Platforms for Applications in Microwave-Accelerated Techniques”**

Muzaffer Mohammed**,** Travis C. Clement and Kadir Aslan

Morgan State University, Department of Chemistry, Baltimore MD 21251.

Corresponding Author: [Kadir.Aslan@morgan.edu](mailto:Kadir.Aslan@morgan.edu)


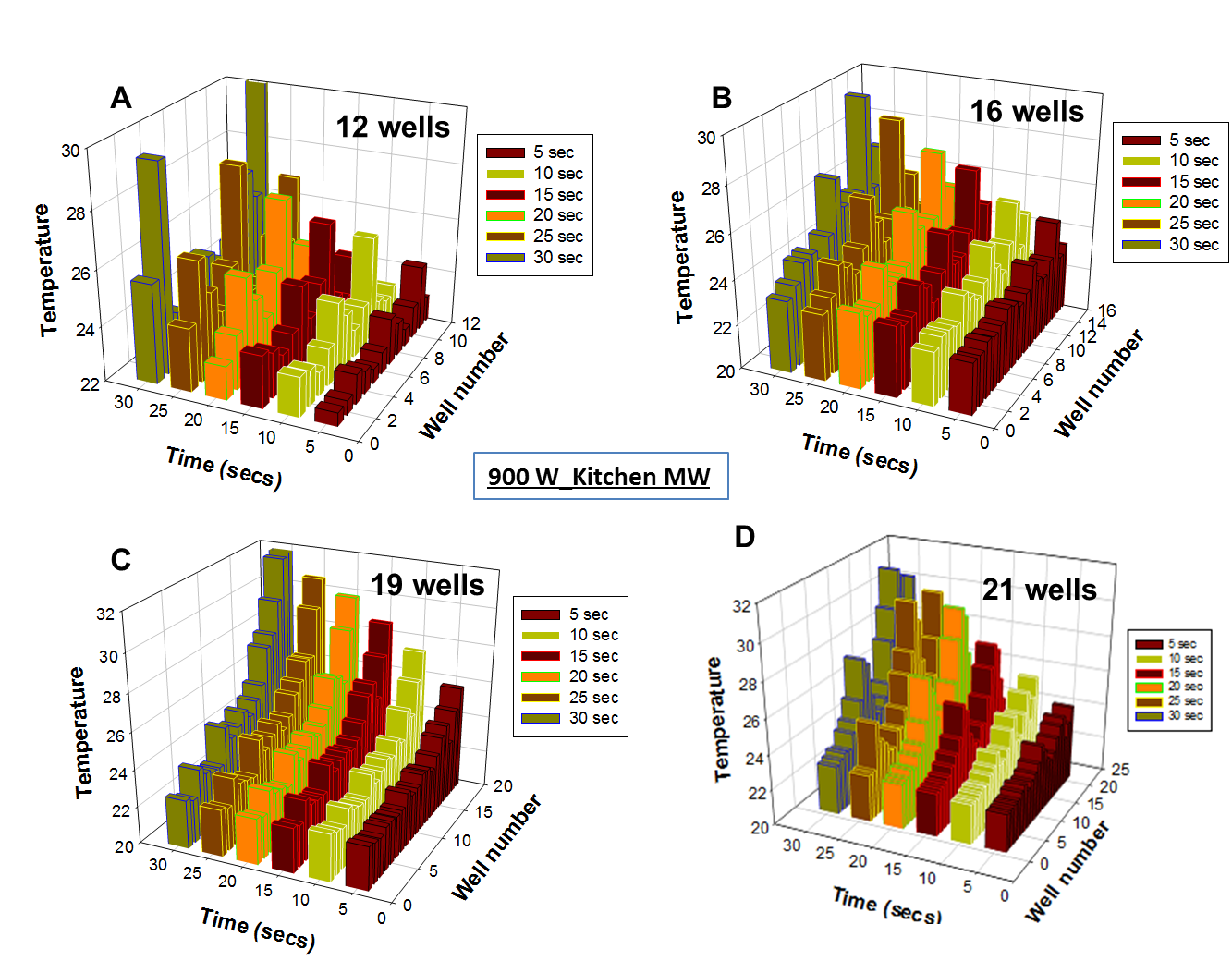


**Figure S1.** Predicted temperature vs. time vs. no of wells graph of **(A)** 12-well **(B)** 16-well and **(C)** 19-well circular bioassay platform in a 900 W kitchen microwave oven.


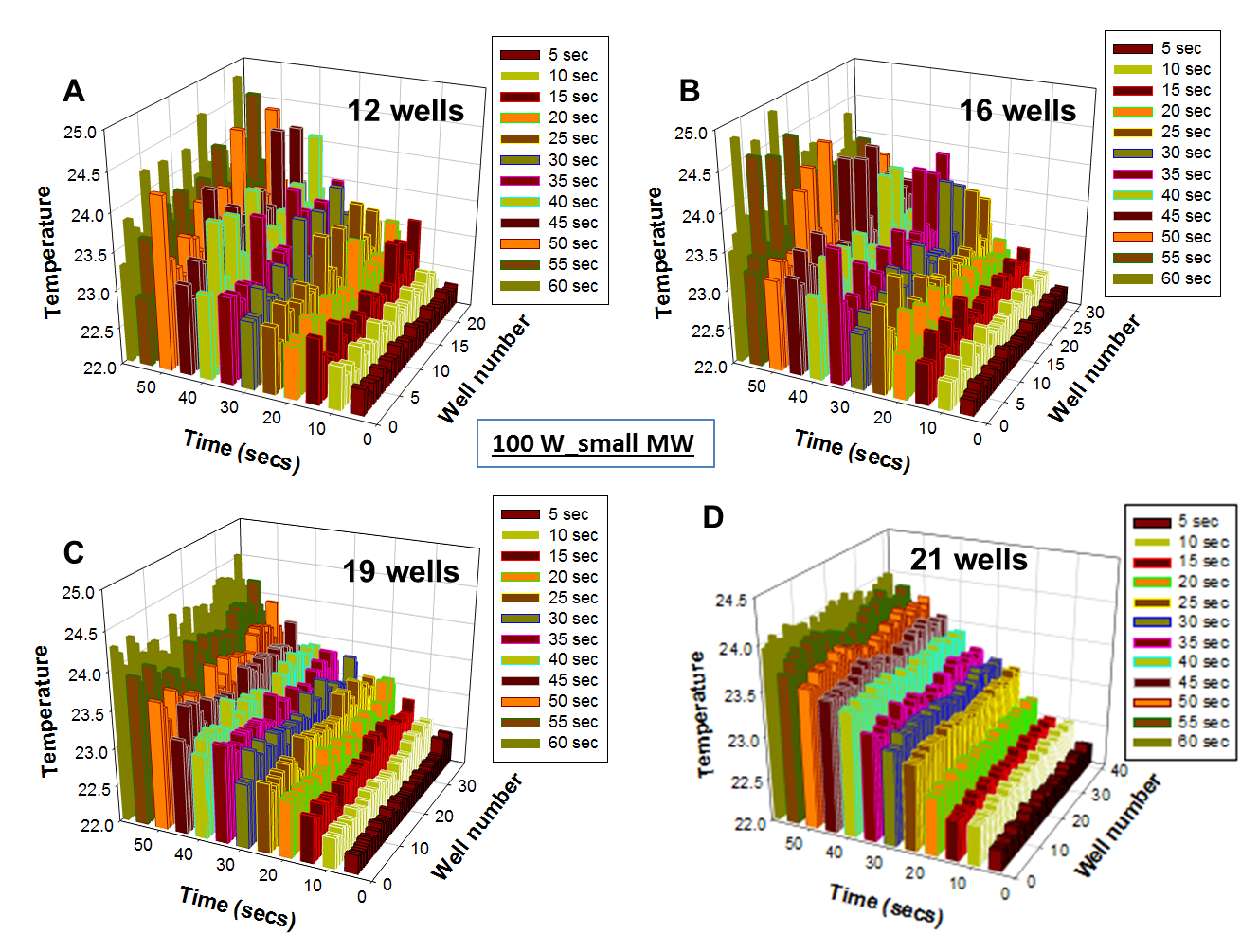


**Figure S2.** Predicted temperature vs. time vs. no of wells graph of two **(A)** 12-well **(B)** 16-well and **(C)** 19-well circular bioassay platform in our microwave cavity.


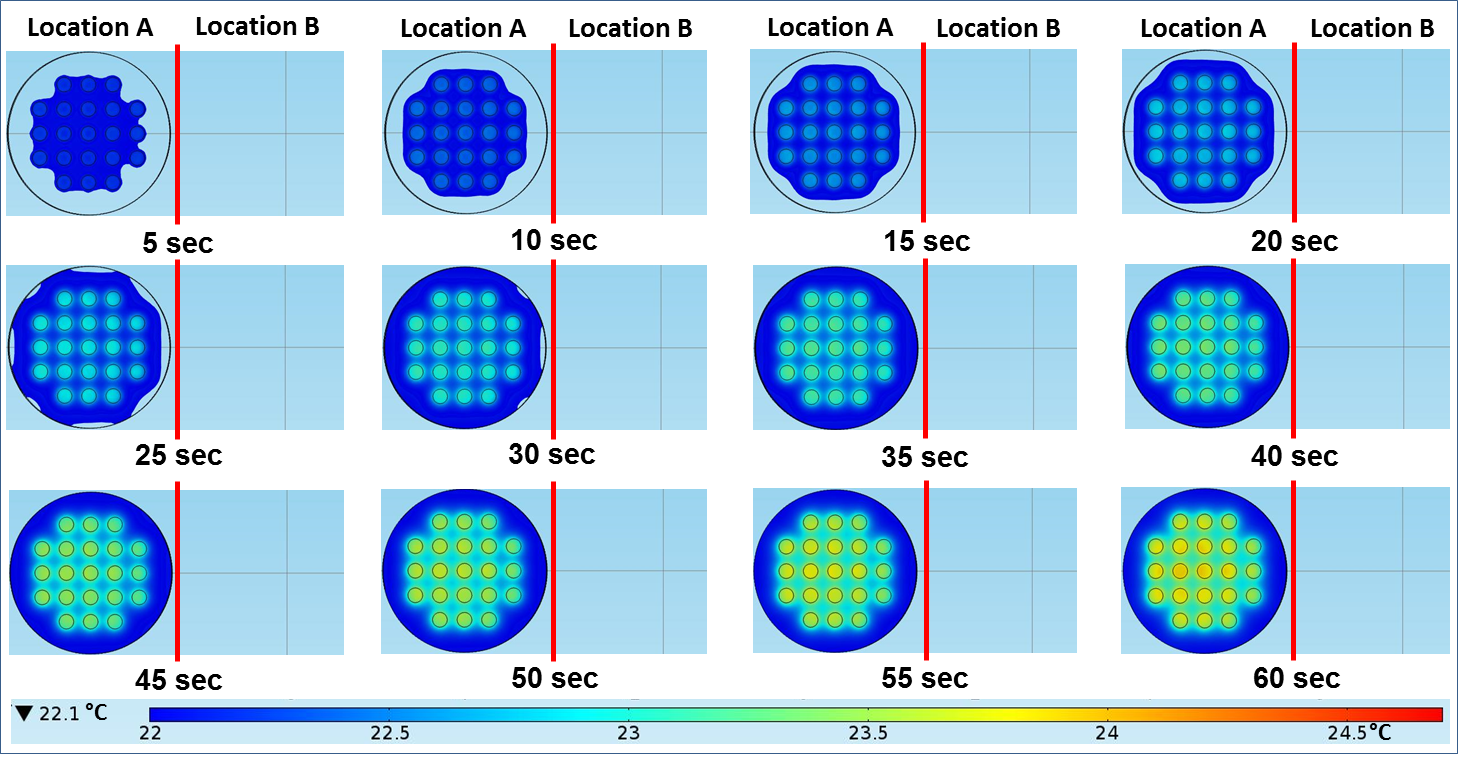


**Figure S3.** Timed images of heating profile of one 21-well bioassay platform at location A in monomode microwave cavity using 100 W power for 60 sec.


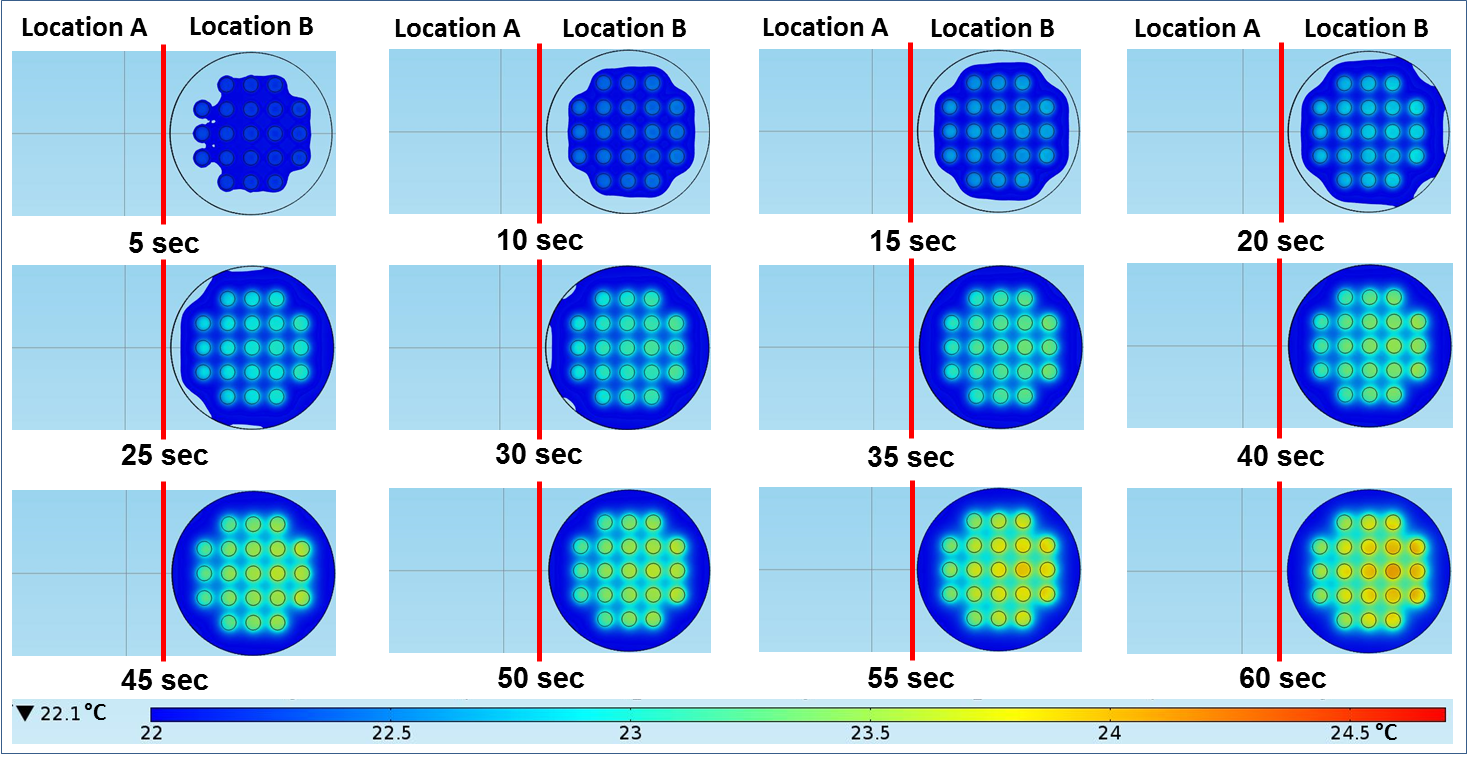


**Figure S4.** Timed images of heating profile of one 21-well bioassay platform at location B in monomode microwave cavity using 100 W power for 60 sec.


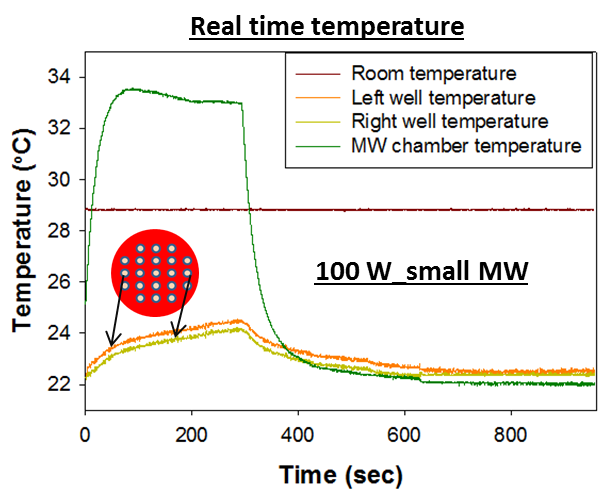


**Figure S5.** Real-time temperature measurement of selected wells of 21-well circular bioassay platform by fiber optic sensor system in our small monomode microwave cavity powered by a 100 W external microwave source. Room temperature refers to air inside the microwave cavity.
